# Is the Dumping Syndrome a Problem After Standard Pancreatoduodenectomy?

**DOI:** 10.1155/1990/85284

**Published:** 1990

**Authors:** Michael Trede

**Affiliations:** Chirurgische Klinik Klinikum Mannheim University of Heidelberg Germany


*HPB Surgery*, 1990, Vol. 2, pp. 77–79
Reprints available directly from the publisher
Photocopying permitted by license only
© 1990 Harwood Academic Publishers GmbH
Printed in the United Kingdom

### HPB INTERNATIONAL

**EDITORIAL & ABSTRACTING SERVICE**

JOHN TERBLANCHE, EDITOR
Department of Surgery,
Medical School · Observatory 7925
Cape Town · South Africa
Telephone: Department Office: 47-1250
Hospital Connection: 47-3311
Telex: 5-22208
Tel. Add: ALUMNI CAPETOWN
Telefax: (021) 47-8955

### IS THE DUMPING SYNDROME A PROBLEM AFTER STANDARD PANCREATODUODENECTOMY?

ABSTRACT

*Linehan IP, Russell RCG and Hobsley M (1988) The dumping syndrome after pancreatoduodenectomy. Surgery, Gynecology & Obstetrics,* 167:114–118

**Some patients have postoperative nutritional problems after a pancreatoduodenectomy. These problems have been attributed without objective evidence to the partial gastrectomy that is performed at the time of the pancreatoduodenectomy. The dumping syndrome has been implicated, and the results of this study determine, for the first time, the role of the dumping syndrome in pancreatic surgical procedures. Sixty-four dumping provocation tests have been performed upon patients with pancreatic disease or after pancreatic surgical treatment. Three patients had the dumping syndrome, and in eight, the result of the test was equivocal.**

**Results of the present study demonstrate an incidence of dumping syndrome after pancreatoduodencetomy of 10%; however, in none of these patients, was the dumping syndrome a significant problem. There was no instance of the dumping syndrome after pylorus-preserving or duodenum-preserving pancreatectomy. It is concluded that, contrary to previous assumptions, the dumping syndrome does not contribute to long term postoperative problems after pancreatic surgical procedures.**

By Permission of *Surgery, Gynecology & Obstetrics.*

### DISCUSSION

KEYWORDS: Pancreatoduodenectomy, dumping syndrome

Standard pancreatoduodenectomy (the Whipple procedure) has been criticized as a "mutilating" procedure in which vital organs such as the gastric antrum and pylorus are being sacrificed unnecessarily. Furthermore, this operation is held responsible for various functional disturbances owing to loss of the pylorus, duodenum, duodenal secretions and the entero-insular axis.

These theoretical disadvantages have so far seemed acceptable in patients with periampullary malignancy, where optimal radicality provides the only chance for cure. However, for chronic pancreatitis involving mainly the proximal part of the organ, less radical procedures have been proposed in recent years: pylorus-preserving pancreatoduodenectomy[6], duodenum-preserving partial proximal pancreatectomy[1] and duodenum-preserving total pancreatectomy[3].

In this paper, the Middlesex Hospital group have for the first time undertaken the task of objectivating as far as possible one alleged functional drawback of the standard Whipple procedure, namely the dumping syndrome. And they did indeed find evidence of dumping in three out of 30 patients submitted either to standard partial or total pancreatoduodenectomy. But in none of these patients was the dumping syndrome a significant problem; in fact these patients had gained weight since the operation. So it would seem that on this count alone, the Whipple operation is perhaps not as deleterious as we were made to believe.

As for the other more "gentle" procedures mentioned above, no hard evidence (certainly not from any controlled trial) has so far been presented that their theoretical advantages have actually paid off in measurable clinical facts. A fair assessment of the situation is formulated by Longmire's group from UCLA when they state that "pyloric-preserving pancreatoduodenectomy is at least functionally equivalent to the standard Whipple resection"[2].

What is more, recently a note of caution has been sounded concerning pylorus-preserving pancreatoduodenectomy. In cancer patients there is some evidence that the potential chance for a curative resection might after all be compromised in some patients[5].

In a large, albeit retrospective comparison between pylorus-preserving and standard Whipple resections, the Mayo Clinic group found the advantages (especially concerning post-operative marginal ulcers) to lie with the Whipple operation[4].

So, in conclusion, it must be left to the personal experience of each surgeon, which type of pancreatoduodenectomy is preferred. The pylorus-preserving procedure may be easier and less time-consuming in expert hands, but until the contrary is proven, it is perfectly in order to adhere to the more radical Whipple operation as regards both the immediate and the long-term functional results.

Michael Trede

Chirurgische Klinik
Klinikum Mannheim
University of Heidelberg
West Germany

### REFERENCES

1. Beger, H.G., Krautzberger, W., Bittner, R., Buchler, M., Limmer, J. (1985) Duodenum-preserving resection of the head of the pancreas in patients with severe pancreatitis. *Surgery*, **97**, 467–473.
2. Fink, A.S., DeSouza, L.R., Mayer, E.A., Hawkins, R., Longmire, W.P., Jr. (1988) Long-term evaluation of pylorus preservation during pancreaticoduodenectomy. *World J. Surg.* **12**, 663–670.
3. Lambert, M.A., Linehan, I.P., Russell, R.C.G. (1987) Duodenum-preserving total pancreatectomy for end stage chronic pancreatitis. *Br. J. Surg.* **74**, 35–39.
4. McAfee, M.K., van Heerden, J.A., Adson, M.A. (1989) Is proximal pancreatoduodenectomy with pyloric preservation superior to total pancreatectomy? *Surgery*, **105**, 347–351.
5. Sharp, K.W., Ross, C.B., Halter, S.A., Morrison, J.G. *et al.* (1989)Pancreatoduodenectomy with pyloric preservation for carcinoma of the pancreas: A cautionary note. *Surgery*, **105**, 645–653.
6. Traverso, L.W., Longmire, W.P., Jr. (1978) Preservation of the pylorus in pancreaticoduodenectomy. *Surgery, Gynecol. & Obstet.*, **146**, 959–962.

